# A randomized trial of chlorhexidine gluconate on oral bacterial pathogens in mechanically ventilated patients

**DOI:** 10.1186/cc7967

**Published:** 2009-07-15

**Authors:** Frank A Scannapieco, Jihnhee Yu, Krishnan Raghavendran, Angela Vacanti, Susan I Owens, Kenneth Wood, Joseph M Mylotte

**Affiliations:** 1 Department of Oral Biology, School of Dental Medicine, University at Buffalo, The State University of New York, 3435 Main St. Buffalo, NY 14214, USA; 2 Department of Biostatistics, School of Public Health and Health Professions, The State University of New York, 3435 Main Street, Buffalo, NY 14214, USA; 3 Department of Surgery and Anesthesiology, School of Medicine and Biomedical Sciences, The State University of New York, 3435 Main Street, Buffalo, NY 14214, USA; 4 Erie County Medical Center, 462 Grider Street, Buffalo, NY 14215, USA; 5 Frontier Science and Technology Research Foundation, 4033 Maple Road, Amherst, NY 14266, USA; 6 Department of Medicine, School of Medicine and Biomedical Sciences, The State University of New York, 3435 Main Street, Buffalo, NY 14214, USA; 7 Current address: Department of Surgery, University of Michigan, 1C421 University Hospital, SPC 50331500 E. Medical Center Drive, Ann Arbor, MI 48109, USA

## Abstract

**Introduction:**

Dental plaque biofilms are colonized by respiratory pathogens in mechanically-ventilated intensive care unit patients. Thus, improvements in oral hygiene in these patients may prevent ventilator-associated pneumonia. The goal of this study was to determine the minimum frequency (once or twice a day) for 0.12% chlorhexidine gluconate application necessary to reduce oral colonization by pathogens in 175 intubated patients in a trauma intensive care unit.

**Methods:**

A randomized, double-blind, placebo-controlled clinical trial tested oral topical 0.12% chlorhexidine gluconate or placebo (vehicle alone), applied once or twice a day by staff nurses. Quantitation of colonization of the oral cavity by respiratory pathogens (teeth/denture/buccal mucosa) was measured.

**Results:**

Subjects were recruited from 1 March, 2004 until 30 November, 2007. While 175 subjects were randomized, microbiologic baseline data was available for 146 subjects, with 115 subjects having full outcome assessment after at least 48 hours. Chlorhexidine reduced the number of *Staphylococcus aureus*, but not the total number of enterics, Pseudomonas or Acinetobacter in the dental plaque of test subjects. A non-significant reduction in pneumonia rate was noted in groups treated with chlorhexidine compared with the placebo group (OR = 0.54, 95% CI: 0.23 to 1.25, *P *= 0.15). No evidence for resistance to chlorhexidine was noted, and no adverse events were observed. No differences were noted in microbiologic or clinical outcomes between treatment arms.

**Conclusions:**

While decontamination of the oral cavity with chlorhexidine did not reduce the total number of potential respiratory pathogens, it did reduce the number *of S. aureus *in dental plaque of trauma intensive care patients.

**Trial Registration:**

clinicaltrials.gov NCT00123123.

## Introduction

The pathogenesis of ventilator-associated pneumonia (VAP) involves aspiration of bacteria from the oropharynx into the lung, and subsequent failure of host defenses to clear the bacteria resulting in development of lung infection [1]. In mechanically ventilated, intensive care unit (MV-ICU) patients, the major potential respiratory bacterial pathogens (PRPs) include *Staphylococcus aureus, Pseudomonas aeruginosa*, *Acinetobacter *species, and enteric species. Previous studies have shown that dental plaque and the oral mucosa are often colonized by PRPs [2,3]. Also, PRPs from dental plaque of MV-ICU patients are genetically identical to strains from bronchoscopic cultures taken at the time pneumonia was suspected, [4,5]. These findings suggest that dental plaque may be an important reservoir of PRPs that cause VAP. Thus, improving oral hygiene in MV-ICU patients and reducing dental plaque load on teeth has the potential to reduce the risk of VAP.

Topical oral application of antiseptics such as chlorhexidine gluconate (CHX) have been evaluated for the prevention of VAP. CHX is a cationic chlorophenyl bis-biguanide antiseptic that has long been approved for use as an inhibitor of dental plaque formation and gingivitis [6-8]. CHX is of particular interest as an oral disinfectant in MV-ICU patients because of its substantivity (the ability of CHX to bind to oral tissues with subsequent slow release and thus a relatively long period of action). Several recently published clinical trials of intra-oral disinfection with topical CHX [9-15] or povidone-iodine gargle and tooth-brushing [16] have demonstrated a reduction in the prevalence of oropharyngeal colonization by PRPs, as well as a reduction in the rate of VAP in MV-ICU patients. Based on these observations, recommendations for preventing VAP have included improving oral hygiene in MV-ICU patients [17,18]. However, not all studies of the use of CHX have shown a reduction in the incidence of pneumonia [19]. Moreover, the studies that have been published used different CHX dosing regimes and did not always clearly define the method of application of CHX. Identification of the minimum frequency for CHX application required to reduce the number of PRPs on the teeth may promote the routine use of this intervention for MV-ICU patients. However, the most simple method for application and the minimally effective dosing regime of CHX that improves oral hygiene to reduce the number of PRPs in dental plaque biofilms has not been determined.

Thus, the goal of the present study was to determine the minimal frequency of oral CHX application (once or twice a day), compared with placebo, which significantly reduces oral colonization by PRPs in MV-ICU patients. Secondary endpoints included incident VAP, duration of mechanical ventilation, length of ICU stay, and rate of mortality.

## Materials and methods

### Patient population

This trial was approved by the University at Buffalo Institutional Review Board. Subjects for this study were recruited from patients admitted to the 18-bed trauma ICU of the Erie County Medical Center (ECMC) who were mechanically ventilated. ECMC is a 550-bed inpatient level 1 regional center for trauma, burn care, and rehabilitation and is an affiliated teaching facility for the State University of New York at Buffalo. This ICU is 'closed', in that all patients who are admitted are primarily managed by the study intensivist physician (KR) or his surrogates. The average length of stay in this unit in the year before the start of the study was approximately six days. Over the past five years the incidence of VAP has ranged from 10 to 50% of all MV patients, and VAP per 1000 ventilator days has ranged from 8 to 12. Once participant eligibility was established, written informed consent was obtained from each patient, or most often from his/her next of kin or health care proxy.

### Sample size estimate

Based on previous studies, it was conservatively estimated that approximately 50% of all subjects admitted to the ICU would be colonized by a PRP. In order to have a 90% power of detecting a difference between colonization proportions, and assuming a dose-response effect whereby 50% of the placebo group would be presumed to be colonized, with 25% of the twice daily CHX group, and 20% of the once daily CHX group, it was determined that a minimum group size would require 53 participants per treatment arm.

### Inclusion/exclusion criteria

Eligible patients were those admitted to the ICU who were expected to be intubated and mechanically ventilated within 48 hours of admission, with the exception of those demonstrating the following exclusion criteria: a witnessed aspiration (to eliminate patients with chemical pneumonitis); a confirmed diagnosis of post-obstructive pneumonia (e.g. advanced lung cancer); a known hypersensitivity to CHX; absence of consent; a diagnosed thrombocytopenia (platelet count less than 40 and/or a INR above 2, or other coagulopathy); a do not intubate order; children under the age of 18 years; pregnant women; legal incarceration; transfer from another ICU; oral mucositis; immunosuppression (either-HIV or drug induced (e.g. organ transplant patients or those on long term steroid therapy)); and re-admission to the ICU.

### Trial design

**Figure 1:**
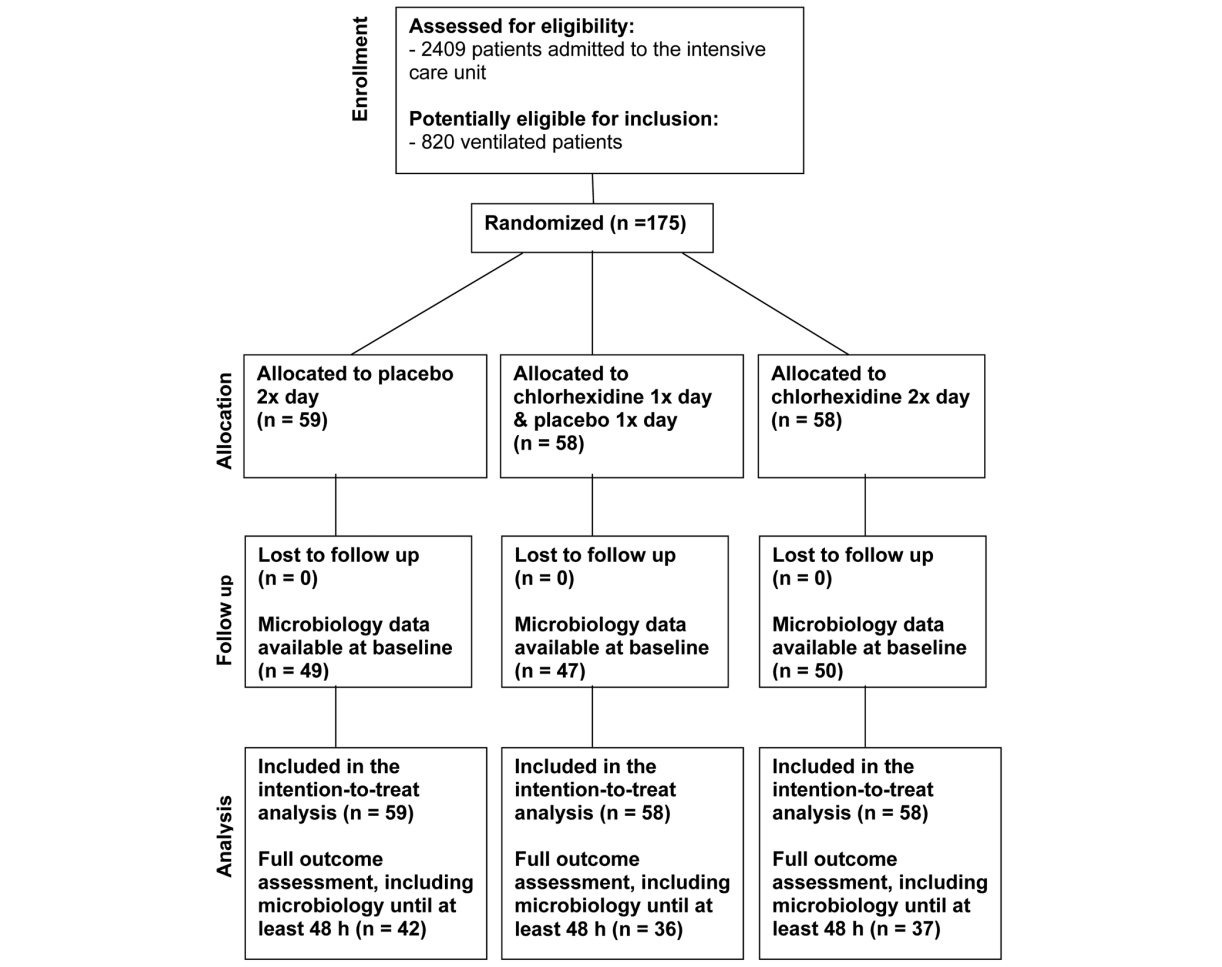
Study design. The final analysis was based on subjects having a microbiologic assessment at baseline and for at least the 48 hour (h) time point (n = 42 in the placebo group, 36 in the chlorhexidine gluconate (CHX) once a day group, and 37 in the CHX twice a day group).

Eligible patients, after giving informed consent and following baseline assessment, were randomly assigned to one of three arms (Figure 1): 1) a control arm in which patients received twice daily oral topical applications (AM and PM) with the CHX vehicle control alone (having the same color, taste, and smell as the CHX rinse); 2) an experimental arm in which patients received once daily oral topical treatment with 0.12% CHX and once daily oral topical treatment with vehicle control (subjects were randomly selected to receive CHX in the morning and placebo in the evening, or CHX in the evening and placebo in the morning); and 3) an additional experimental arm in which patients received twice daily oral topical treatments with 0.12% CHX gluconate.

### CHX formulation

The oral rinse was formulated [20] as follows: 3 ml chlorhexidine gluconate 20% added to 200 ml of distilled water; separately 5 ml essence of peppermint was mixed with 5 ml 95% ethanol, and then 15 ml glycerin. The solutions were then mixed and brought to 500 ml with distilled water. The placebo contained all ingredients, except for CHX, which was substituted with distilled water.

### Concealment, blocking, and randomization

Subjects were randomized to the study via a web-based subject enrollment system that used protocol-specific specification files that presented questions to site personnel to evaluate eligibility. Only subjects who met all eligibility requirements were randomized to the study. The randomization system prepared a set of Subject Identification Numbers (SID) that identified individual treatment assignments. The study nurse obtained the SID number based on the randomization when the subject was enrolled. The assigned SID number was sent to the study pharmacist to dispense the appropriate blinded treatment. Assignment of treatment was blinded to patients and all investigators including outcome assessors, statisticians, and care providers. Sealed envelopes containing a random number were generated in blocks of six to provide concealment of patient assignment from the investigators. Each block of six numbers were assigned corresponding boxes prepared and numbered sequentially from one to six. The pharmacists prepared each box to contain two 32 oz bottles, one labeled AM and one labeled PM. For each block one of 720 possible permutations of symbols (Placebo 1(P_1_), Placebo 2 (P_2_), Once AM 1 (OM_1_), Once PM 1 (OM_2_), Twice a day 1 (T_1_), Twice a day 2 (T_1_)) were selected using a random number generator, and numbers one to six were assigned based on randomly selected permutations. The oral topical treatment for each box was formulated and prepared by the hospital pharmacy.

### Intervention

CHX was applied using a rinse-saturated oral foam applicator (Sage Products, Cary, IL, USA) twice each day (in the morning at about 8 AM and in the evening at about 8 PM). Applications were for one minute with about 1 oz of 0.12% CHX or placebo. The ICU staff nurses performed CHX application. All teeth, the oral soft tissues including buccal mucosa, vestibule, gingiva, and the floor of the mouth and tongue dorsum were swabbed. Excess rinse was suctioned out of the subject's mouth after one minute. In addition to this rinse, the routine oral care regime used by the ICU was maintained throughout the course of the trial. Briefly, routine oral care included use of a suction toothbrush (Sage Products, Cary, IL, USA) twice a day and as needed to brush teeth and the surface of the tongue, for approximately one to two minutes, and applying suction at completion and as needed during the brushing. In addition, swabbing with Peroxamint solution (Sage Products, Cary, IL, USA) was performed every four hours. This was followed by the application of a mouth moisturizer (Sage Products, Cary, IL, USA) to the oral mucosa using a fresh swab. Also, deep suctioning was performed to assist in removing oropharyngeal secretions pooled on top of the cuff of the endotracheal tube every 12 hours and following position changes.

The study nurses trained all ICU staff nurses to perform the standardized technique for application of the rinse. The study nurses periodically observed ICU staff nurses for adherence to study protocol. Routine in-service sessions were held every three months to review the protocol with ICU staff nurses. The study nurses collected all samples and study data and followed each enrolled subject until they were exited from the study.

### Dental plaque and airway sampling

Dental plaque samples were collected on the day of admission to the ICU (baseline sampling) and every 48 hours thereafter until discharge from the study. Samples consisted of supragingival dental plaque pooled from at least two remaining teeth, or if the patient was edentulous, from the buccal mucosa, or, if they were wearing a denture, from the tissue surface of the maxillary denture. Samples were collected with a sterile stainless steel curette and dispersed into 2 ml of sterile normal saline. For patients who were edentulous and not wearing dentures, the buccal mucosa was sampled by vigorous rubbing with a sterile swab and dispersed into sterile normal saline (2 ml). Sampling was performed prior to routine daily oral care. Samples were immediately sent to the laboratory for processing.

Tracheal samples were obtained from all intubated patients at each sampling interval (baseline and every 48 hours) using a sterile suction catheter placed into the endotracheal tube. When pneumonia was suspected (see below), lung secretions were obtained and analyzed for bacterial infection by Blind Quantitative Bronchoalveolar Lavage (bqBAL) using a mini-BAL technique.

### Microbiological procedures

Samples were vortexed for one minute and split into 4 to 0.5 ml portions (with three of the samples placed at -70°C). The remaining dental plaque samples were diluted and plated using a spiral plater on sheep's blood agar (to isolate *S. aureus*), and MacConkey agar (for isolation of Gram-negative bacilli). For tracheal secretions, semi-quantitation of PRPs was performed (0 to 4+ range scored). Microbial cultures were incubated for 72 hours at 37°C in 5% carbon dioxide. Plates were assessed for growth of the following target PRPs: *S. aureus, P. aeruginosa, Acinetobacter *species, and enteric organisms (*Klebsiella pneumoniae*, *Serratia marcescens*, *Enterobacter *species, *Proteus mirabilis*, *Escherichia coli*). Results of quantitative cultures were expressed as colony forming units (cfu) per ml. A bqBAL sample was considered positive if 10^4 ^CFU/ml or more of a PRP was found.

For measurement of CHX resistance, dental plaque and lower airway secretions processed for microbiologic analysis as described above, were plated on trypticase soy agar with and without a final concentration of 0.12% CHX gluconate. Plates were incubated both aerobically and anaerobically for 72 hours and the proportion of CHX-resistant colonies enumerated.

### Patient data, outcome variables, and potential confounding variables

All subjects entered into the study were followed for up to 21 days, or on discharge from the ICU, extubation, or death. Although it was uncertain when planning the trial that enough subjects could be recruited to discern a statistically significant effect of the oral intervention on incident VAP, we selected as primary outcome variables dental plaque score and colonization of the oral cavity by respiratory pathogens (*S. aureus, P. aeruginosa, Acinetobacter *species, and the enteric species *K. pneumoniae, S. marcescens, Escherichia *species, *P. mirabilis*, and *E. coli*) by quantitative culture measured at baseline and subsequently at 48-hour intervals until the subject was discharged from the ICU.

Secondary outcomes assessed were: diagnosis of pneumonia in a MV patient at least 48 hours following admission to the ICU (see below); mortality following admission and during the stay in the ICU; length of ventilation in the ICU; and length of stay in the ICU.

The clinical pulmonary infection score system (CPIS) used is based on five different elements: partial pressure of arterial oxygen (PaO_2_)/fraction of inspired oxygen (FiO_2_); Infiltrate on chest radiograph; Leukocytosis; purulent secretions; and fever. The CPIS score was calculated as follows: 1) Fever: 0 (36.5 to 38.4°C), 1 (38.5 to 39), 2 (<36.0 OR >39.0); 2) Leukocytosis: 0 (4000 to 11,000 wh.ite blood cells per mm^3 ^of blood), 1 (11,000 to 17,000), 2 (>17,000); 3) New infiltrate: 0 = None, 1 = Patchy, 2 = Localized; 4) Secretions: 0 = None to minimal, 1 = moderate, 2 = large amount; and 5) PaO_2_/FiO_2_: 0 = more than 330 and 2 = less than 330.

A CPIS score 6 or more triggered sampling of the lower airway by bqBAL using a Combicath^® ^(KOL Bio Medical Instruments, Chantilly, VA, USA). Briefly, this technique involves insertion of the Combicath through the endotracheal tube and its advancement until resistance was encountered [21,22]. The catheter was then withdrawn by a cm, the inner portion of the co-axial catheter advanced to dislodge the poly-ethylene glycol plug, and then 50 ml of normal saline was instilled through the catheter. After 30 seconds, the specimen was withdrawn and sent for quantitative bacteriology. The presence of 10^4 ^cfu/ml or more of a target PRP in bqBAL fluid or a positive pleural fluid culture in the absence of previous pleural instrumentation was considered as positive evidence for a diagnosis of pneumonia.

The following demographic information was recorded: age; gender; race; admission diagnosis; admitted from i) community, ii) hospital ward, iii) other ICU, or iv) nursing home.

The following variables were assessed following patient discharge: total days of hospital admission; type of antibiotic therapy (within 14 days), and total duration in days prior to admission to the ICU.

The severity of illness score utilizing the Acute Physiology and Chronic Health Evaluation (APACHE) II system was determined for each subject at baseline [23]. This index utilizes information obtainable from the patient's hospital record, including physiologic information (temperature, mean arterial pressure, heart prevalence, respiratory prevalence, oxygenation, arterial pH, serum levels of Na, K and creatinine, hematocrit, white blood count), and age.

### Oral examination

The study nurse performed a baseline oral examination. Follow-up oral examination and clinical sample collection was performed every 48 hours until the patient was discharged from the study. The oral examination consisted of a simplified plaque index and the enumeration of missing teeth. The plaque index was performed for six teeth (the upper right first molar, upper right central incisor, upper left first bicuspid, lower left first molar, lower left central incisor, and lower right first bicuspid). A Michigan O-probe and oral mirror were used to assess plaque. Only the mesial-buccal and mesial-lingual surfaces at the gingival margin of the teeth were scored. The amount of plaque present on the tooth was scored 0 to 3 as follows: 0 = no plaque noted; 1 = plaque seen only on the tip of an explorer passed over the tooth surface; 2 = plaque obvious with the naked eye; 3 = gross deposits of plaque present over the entire tooth.

The plaque index was calculated as the average of the scores obtained from the six teeth. For those patients missing any of the index teeth, or those with endotracheal tube placement and/or security that precluded examination, teeth available to be examined closest to the missing teeth were scored. Only a single score was given for each tooth, representing the surface harboring the most plaque.

The hard-palate, soft palate, buccal mucosa, tongue, and gingiva were examined for abnormalities, including inflammation, ulceration or other signs of inflammatory irritation that might be expected to be secondary to exposure to CHX.

### Adverse events

All subjects were monitored for potential adverse events, which included intraoral events (mucositis, thrush, tooth staining, alterations in taste, tooth hypersensitivity) and systemic adverse events (mortality).

### Data management

Once subjects were enrolled to the study site, study personnel completed protocol specific case report forms according to the study evaluation schedule. The case report forms were entered into the study database via a distributed data entry system.

Computerized data checks were run on the data as it was entered into the database. These computerized checks were tracked for resolution. The study data manager also ran manual QA checks for inconsistent data. Inconsistent data items were queried by the study data manager, which were followed to resolution.

### Statistical analysis

All tests were carried out using intent-to-treat analysis. All tests were two-sided using a significance level of 0.05. Baseline comparisons between groups were performed using analysis of variance (ANOVA) and/or the chi-squared test, as appropriate. The primary outcome variable, the colonization of the oral cavity by target respiratory pathogens, was observed in each patient until their discharge from the ICU. A mixed effect model was used to compare overall treatment effects between groups for repeated measures data. It is known that, under the assumption that data is randomly missed, a likelihood based method (such as the mixed effect model) provides unbiased inference regardless of missing data. The group comparison for each time point was performed using ANOVA. All secondary continuous variables with repeated measures data were analyzed similarly. The group comparisons with categorical variables were carried out using the chi-squared test. Time to VAP was defined as the interval between the time of enrollment and time of the first diagnosis of pneumonia, as confirmed by a positive bqBAL. Probability plots for time to VAP were estimated by the Kaplan-Meier method. The distributions of time to VAP were compared using the Cox proportional hazards model.

## Results

### Description of study participants

The study design and patient recruitment outcomes are depicted in Figure 1. Initiation of patient recruitment commenced on 1 March, 2004 and ended on 30 November, 2007. A total of 2409 patients were admitted to the ICU during this period. Of these, a total of 820 were ventilated and therefore potentially eligible for inclusion. Patients were admitted to the study who showed no criteria for exclusion, and who, following consultation with the attending physician and nursing staff, would likely remain ventilated for at least 48 hours. Of the 175 patients who initially provided informed consent and who were randomly assigned to one of three groups (placebo – vehicle control, CHX once a day, CHX twice a day), 19 were extubated or expired before sampling. Another 10 subjects were eliminated from the analysis due to missing data, leaving 146 patients having microbiologic data at least at baseline. Thus, analysis of the primary outcomes (dental plaque score and the colonization of target pathogens present in dental plaque) was performed on data from 146 subjects. Of these, 115 were assessed at 48 hours (e.g. microbiologic assessment at the 48 hour time point (n = 42 in the placebo group, 36 in the CHX once a day group, and 37 in the CHX twice a day group), 93 subjects were assessed at 96 hours, 74 subjects at 144 hours, 69 at 192 hours, 57 subjects at 240 hours, 40 subjects at 288 hours, 30 subjects at 336 hours, 38 subjects at 384 hours, 20 subjects at 432 hours, and finally 12 subjects at 480 hours. In addition, intent to treat survival analysis was performed for all 175 patients who were initially randomized.

Demographic and clinical characteristics of the subjects enrolled in this study are presented in Table 1. The median age of the 146 study subjects was 48.0 years (range: 18.0 to 87.5), with the majority being white males admitted from the community. The groups were well balanced with respect to age, gender, APACHE score at baseline, and antibiotic exposure before inclusion in the study (Table 1). Of the 146 subjects, 16 were edentulous: 7 of 49 in the control group, 3 of 47 in the CHX once a day group, and 6 of 50 in the CHX twice a day group (*P *= 0.4449). We found no correlations between co-morbidities and group assignment (Table 1).

**Table 1:** Subject characteristics and outcomes between groups

Variables	Total (n = 146)	Control (n = 49)	CHX*1 (n = 47)	CHX*2 (n = 50)	*P *value
**Age (years)**	48.0 ± 20.8	50.0 ± 22.5	44.8 ± 19.9	47.6 ± 19.1	0.3948
**Gender – male/female**	104/42	36/23	43/15	44/14	0.1570
**Race**					0.3661
**White**	117 (88%)	43 (88%)	37 (78%)	37 (74%)	
**African American**	24 (17%)	4 (8%)	10 (22%)	10 (20%)	
**Asian**	2 (1%)	1 (2%)	0 (0%)	1 (2%)	
**American Indian**	3 (1%)	1 (2%)	0 (0%)	2 (4%)	
**Admitted from**					0.0989
**Internal hospital ward**	6 (4%)	5 (10%)	1 (25)	0 (0%)	
**External community**	116 (80%)	38 (76%)	37 (79%)	41 (82%)	
**Other**	24 (16%)	6 (12%)	9 (19%)	9 (18%)	
**Co-morbidities**	68 (47%)*	24 (50%)	19 (40%)	25 (51%)	0.5212
**Cardiac**	35 (24%)	13 (27%)	10 (21%)	12 (25%)	0.8039
**Pulmonary**	16 (11%)	7 (15%)	3 (6%)	6 (12%)	0.4245
**Renal**	5 (3%)	2 (4%)	0 (0%)	3 (6%)	0.2483
**Hepatic**	2 (1%)	2 (4%)	0 (0%)	0 (0%)	0.1316
**Endocrine**	24 (17%)	10 (21%)	6 (13%)	8 (16%)	0.5715
**Central nervous**	21 (15%)	9 (19%)	8 (17%)	4 (8%)	0.2844
**Neoplastic**	10 (7%)	4 (8%)	2 (4%)	4 (8%)	0.6764
**Immunosuppression**	2 (1%)	0 (0%)	0 (0%)	2 (4%)	0.1400
**APACHE (baseline)**	19.1 ± 5.5	19.1 ± 6.1	18.5 ± 4.1	19.7 ± 6.1	0.5850
**Antibiotic exposure before study inclusion**	43 (29%)	18 (37%)	12 (26%)	13 (26%)	0.3896
**Antibiotic exposure after study inclusion**	103 (71%)	32 (65%)	34 (72%)	37 (74%)	0.6043
**Antibiotic use**	3.5 ± 3.6	3.1 ± 3.5	3.8 ± 3.6	3.7 ± 3.8	0.6057
**Days ventilated**	9.1 ± 5.6	9.7 ± 6.3	9.4 ± 5.0	8.4 ± 5.2	0.5180
**Days in hospital**	11.4 ± 6.6	11.3 ± 6.7	12.0 ± 6.3	11.0 ± 6.8	0.7263
**Deaths**	24 (17%)	8 (17%)	8 (17%)	8 (16%)	0.9834
**Missing doses**	0.7 ± 1.1	0.9 ± 1.2	0.7 ± 1.3	0.5 ± 0.8	0.2844

*Some patients had multi-comorbidities.

APACHE = Acute Physiology and Chronic Health Evaluation; CHX = chlorhexidine gluconate.

### Dental plaque scores and microbiology results

**Figure 2:**
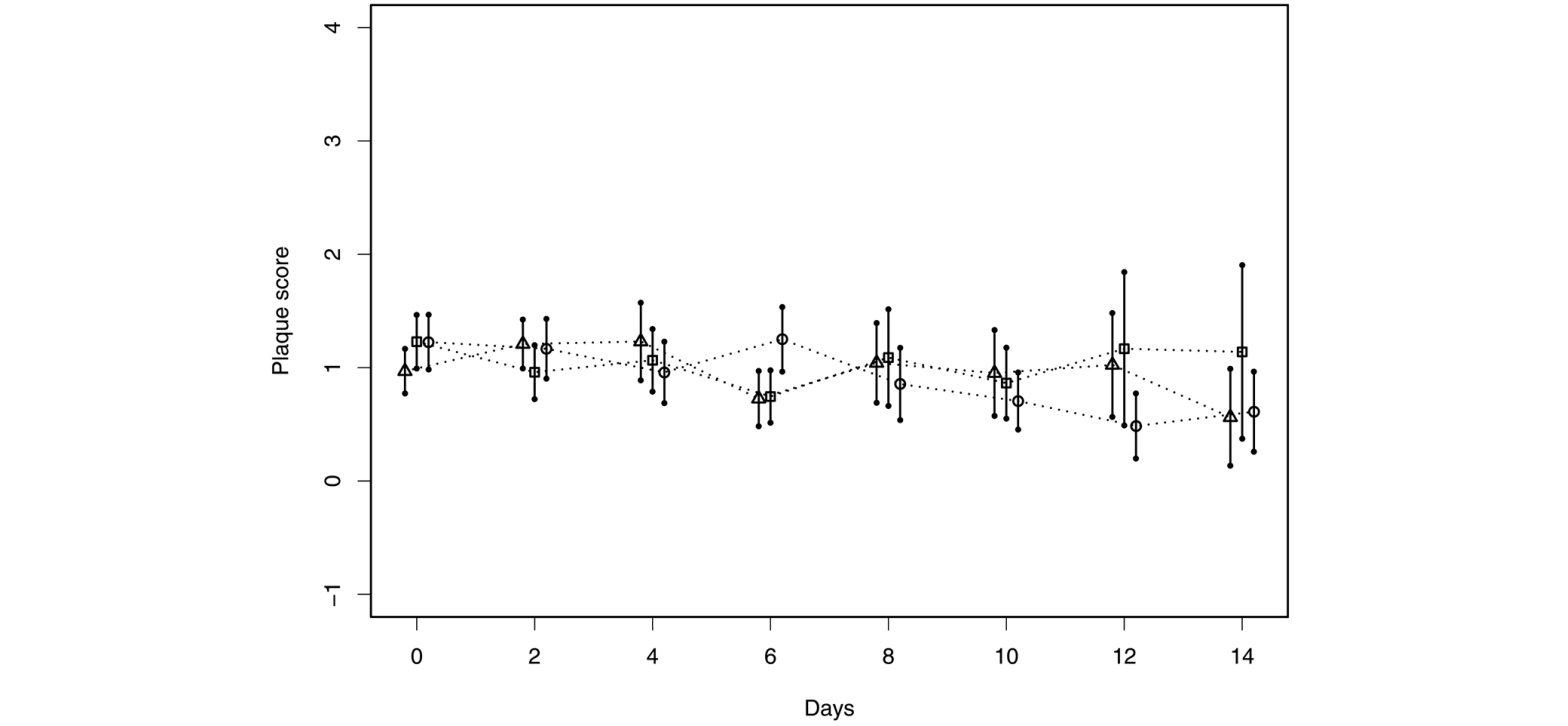
Effect of topical CHX on dental plaque scores (mean) and 95% confidence intervals. Triangle = placebo; Square = chlorhexidine gluconate (CHX) once a day; Circle = CHX twice a day. Days represent duration following mechanical ventilation. The confidence intervals were obtained based on data at each time point.

**Figure 3:**
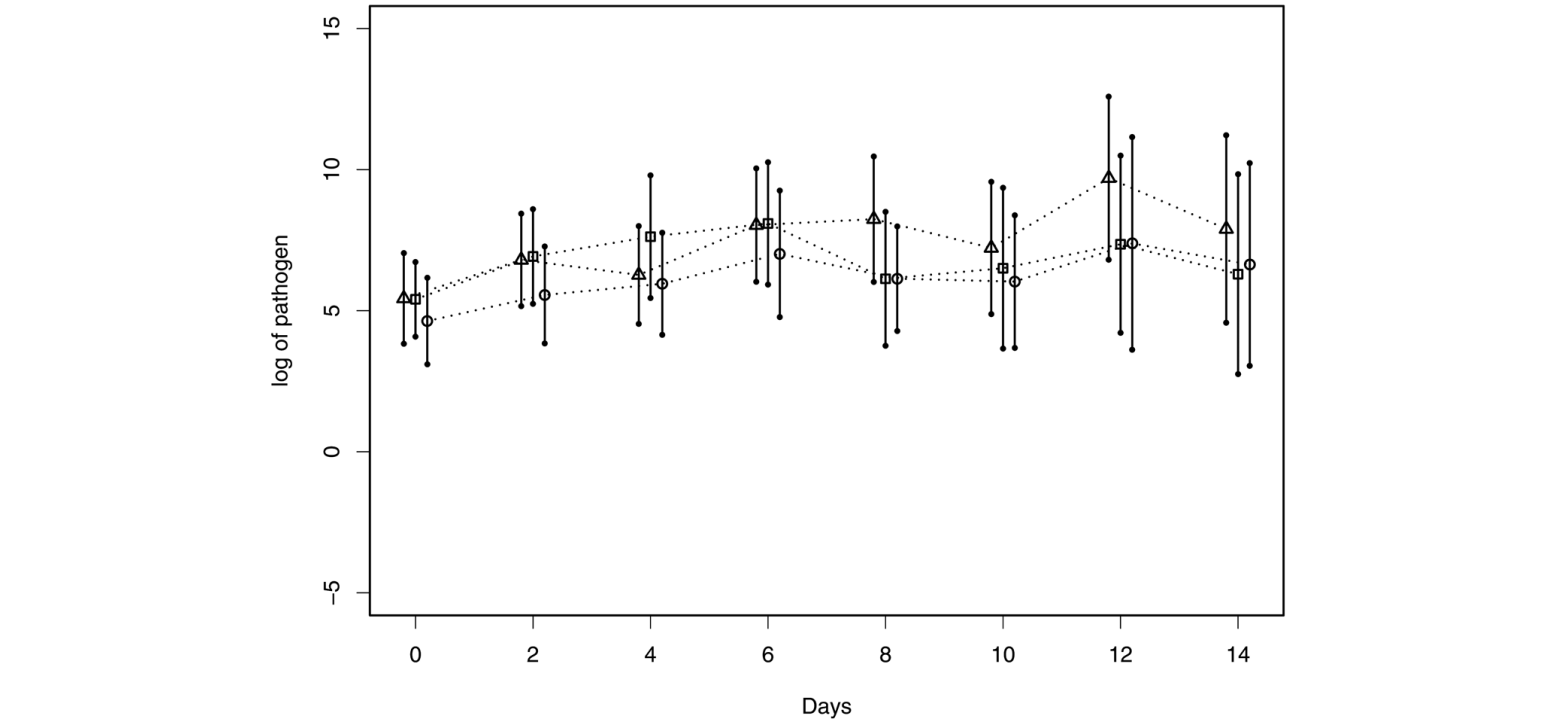
Effect of topical chlorhexidine gluconate on the number of potential respiratory bacterial pathogens in oral samples. Mean in log scale with 95% confidence intervals. The confidence intervals were obtained based on data at each time point. Triangle = placebo; Square = chlorhexidine gluconate (CHX) once a day; Circle = CHX twice a day.

**Figure 4:**
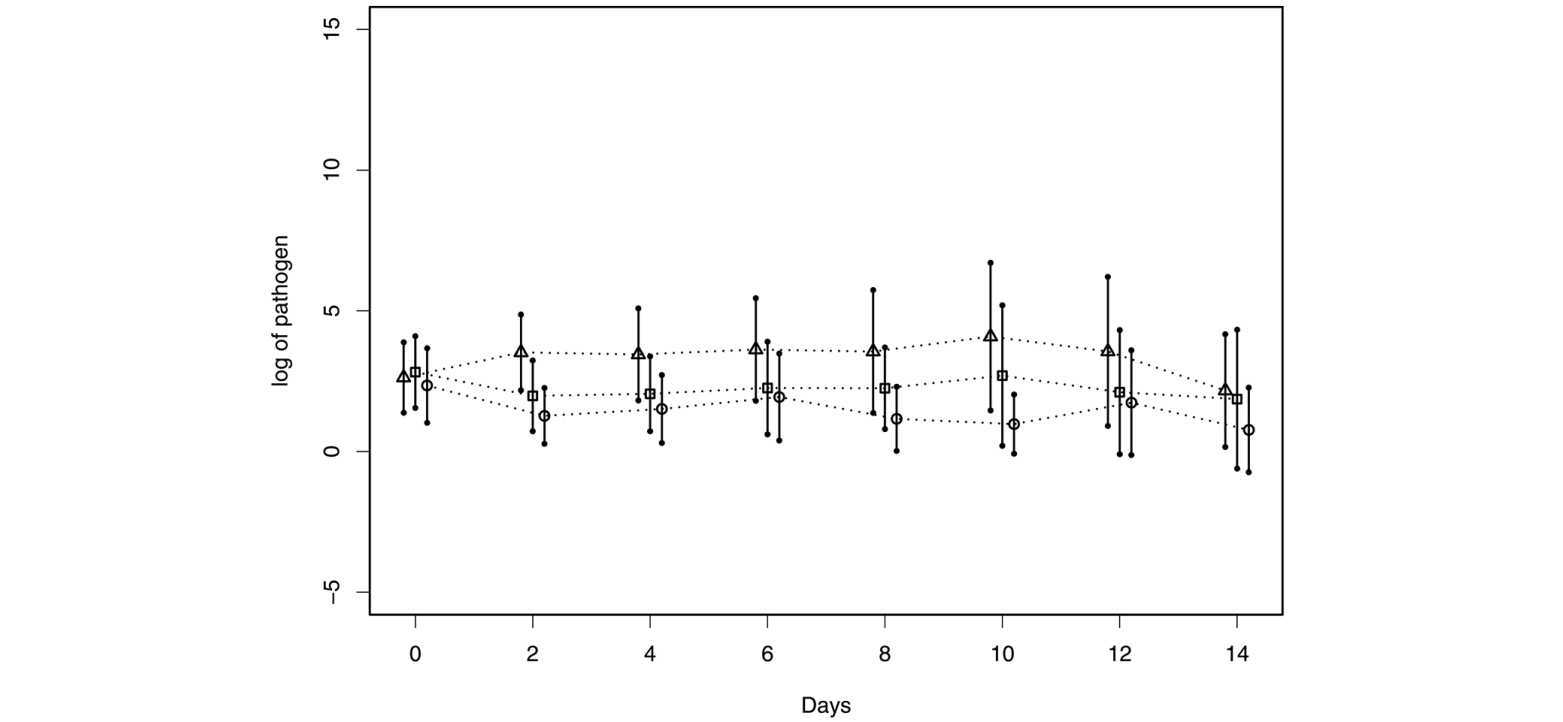
Effect of topical chlorhexidine gluconate on the number of *Staphylococcus aureus *in oral samples. Mean in log scale with 95% confidence intervals. The confidence intervals were obtained based on data at each time point. Triangle = placebo; Square = chlorhexidine gluconate (CHX) once a day; Circle = CHX twice a day.

Neither CHX once a day or twice a day showed a significant reduction in plaque scores when compared with the placebo group (Figure 2, overall treatment effect *P *= 0.8423). CHX delivered once or twice a day yielded similar results with respect to the number of PRPs in dental plaque samples. When compared with the placebo control group, no reduction in total counts or PRPs as a percentage of total cultivable flora were noted between the placebo and either CHX group, at all time points (Figure 3, overall treatment effect *P *= 0.6750). In addition, the total number of cfus recovered as enterics, *Pseudomonas *and *Acinetobacter *were not reduced by CHX at any time point. A statistically significant reduction was, however, noted in the total number of *S. aureus *recovered in the CHX groups when compared with the placebo group (Figure 4, *P *= 0.0065 and 0.0201 at day 2 and day 4, respectively). CHX treatment also did not appear to reduce tracheal colonization by PRPs (data not shown).

Dental plaque samples from the first 100 subjects enrolled into the study were also plated on blood agar plates supplemented to 0.12% CHX. In all cases, these plates showed no growth, demonstrating that the intervention did not select for CHX-resistant bacteria.

### Clinical pulmonary results

**Figure 5:**
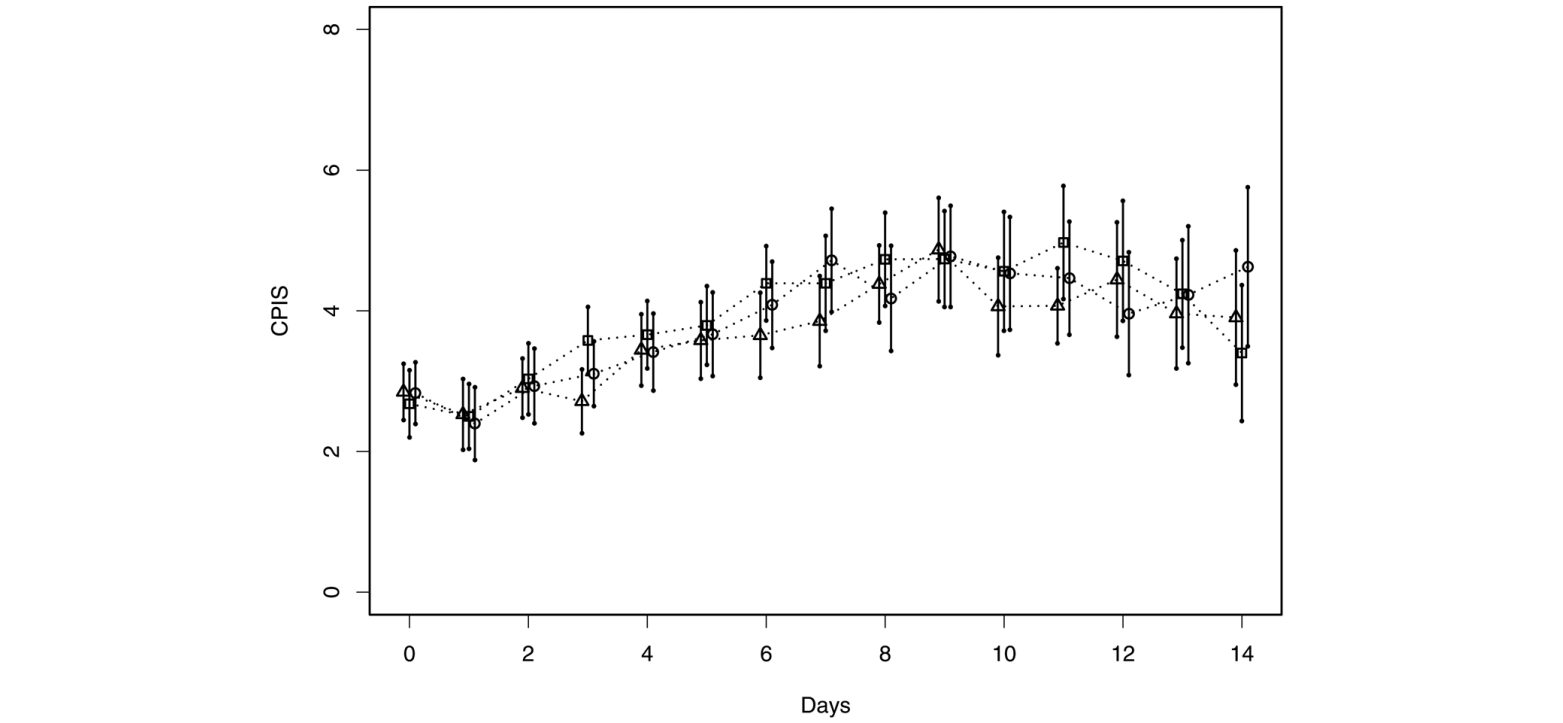
Effect of CHX on mean clinical pulmonary infection score with 95% confidence intervals. Triangle = placebo; Square = chlorhexidine gluconate (CHX) once a day; Circle = CHX twice a day. The confidence intervals were obtained based on data at each time point.

No intra oral adverse events were noted, including mucositis or tooth staining. No significant differences were found between groups (Table 1) with respect to antibiotic use, ventilator days, days in the hospital, mean CPIS score (Figure 5), or mortality. When pneumonia was defined as the presence of more than 10^4 ^cfu of pathogen/ml of bqBAL fluid, among all 175 patients, 12 subjects in the control group were found to have pneumonia; in the CHX once a day group, 7 subjects were diagnosed with pneumonia, while in the CHX twice a day group 7 subjects also had pneumonia. Using intent-to-treat analysis, a 41% of reduction in the rate of pneumonia was noted between the treated and placebo group (odds ratio (OR) = 0.54, 95% confidence interval (CI): 0.23 to 1.25, *P *= 0.1459); however, the differences were found not to be statistically significant (*P *= 0.1459). The incidence of pneumonia by survival analysis showed that the onset of pneumonia tended to be delayed in the treated groups when compared with the control group; however, these differences were not statistically significant (hazards ratio (HR) = 0.555, 95% CI: 0.256 to 1.201, *P *= 0.1348). If patients who were discharged or diagnosed with pneumonia on the day of enrollment into the study (five and one patients, respectively) were excluded, a decreased risk for VAP in the CHX-treated group (HR = 0.514, 95% CI: 0.234 to 1.127, *P *= 0.0966) was observed.

No differences were noted between groups in the number of missed doses of CHX (or placebo) delivered to each subject (Table 1).

## Discussion

As VAP continues to be a common complication of critical care, development of preventive approaches are essential to reduce the incidence of this costly infection. The goal of the present trial was to determine the minimum frequency (once or twice a day) of oral decontamination with 0.12 CHX required to improve oral hygiene and reduce oral colonization by potential respiratory pathogens in intubated MV patients admitted to the trauma ICU. The strengths of this trial include the well-controlled, randomized, blinded, well-concealed, and blocked design, and the fact that the intervention was provided by staff nurses, thus allowing for the test of the intervention under 'real world' conditions.

The results show that the use of oral topical CHX resulted in a quantitative reduction in the number of *S. aureus *cfus in the dental plaque of MV-ICU patients. The finding that CHX treatment reduced the number of *S. aureus *in the oral cavity supports previously published studies that suggest that *S. aureus *is vulnerable to CHX disinfection [14]. However, CHX did not appear to reduce the total number or proportion of other target PRPs in dental plaque (*Pseudomonas, Acinetobacter *or enteric species). Although the group size was not sufficiently large to show a statistically significant reduction in the incidence of VAP between groups, there was a trend for fewer cases of VAP in both CHX groups.

There are several possible explanations to explain why CHX did not reduce the numbers of Gram-negative pathogens in oral biofilms. First, it is known that Gram-positive pathogens such as *S. aureus *are more sensitive to CHX than are Gram-negative pathogens [13,24]. Second, because dental plaque biofilm was the primary oral sample analyzed, it is likely that the organisms cultured from the biofilm were resistant to the topically delivered antimicrobial agent. It is well known that bacteria that adopt a biofilm lifestyle are usually resistant to antimicrobial agents. Such resistance has been attributed to slow growth in the biofilm, the induction of stress responses, and or production of exopolysaccharides or other extracellular components that exclude the antimicrobial agent from the bacterial community within the biofilm [25].

A number of published studies suggest that topical CHX twice a day prevents VAP [11-15,26]. Furthermore, several studies have demonstrated the genetic similarity of bacteria isolated from the lung to bacteria isolated from dental plaque, demonstrating that dental biofilms are an important reservoir for these pathogens [4,5]. Thus, mechanisms other than reduction of PRPs in dental biofilm must be considered to help explain the apparent efficacy of this agent to prevent VAP. One possibility is that CHX inhibits the viability of the planktonic bacteria in the oral secretions. The subsequent reduction in the number of viable PRPs in the secretions thus reduces the number of viable organisms aspirated into the lower airway and therefore will prevent subsequent infection. Alternatively, the virulence potential of the bacteria may be reduced by CHX. Previous studies have suggested that CHX is able to bind to bacterial components such as lipopolysaccharide and proteases [27-29]. Such interactions may diminish the biologic activity of such components to reduce the virulence potential of bacteria. It is also possible that concomitant use of other oral care products such as toothpaste might reduce CHX efficacy [30].

Adverse events have rarely been reported in clinical trials of CHX in ICU patients. A meta-analysis of seven clinical trials found that adverse effects were not reported in any of these studies [31]. A recent clinical trial of 2% CHX versus saline found that 9.8% in the CHX group had mucosal irritation versus 0.9% in saline controls (*P *= .001). The present study using 0.12% CHX found no adverse effects (mucosal irritation or tooth staining).

No previous published clinical trials of CHX in the context of VAP have assessed resistance to this agent during the study. In the present study, an effort was made to identify resistance to CHX by plating samples of dental plaque or lower airway secretions on agar containing 0.12% CHX. Although this approach most likely detects high level CHX resistance, no such resistant bacterial colonies were noted using this method. This finding indicates that resistance to CHX was not likely to be an explanation for the findings of this study.

Previous meta-analyses of trials conclude that this CHX is effective in prevention of VAP [31,32]. These analyses revealed, however, that there was variation in the populations studied as well as in the concentration, preparation, and dosing schedule of CHX. Clinical trials of CHX have tested concentrations of 0.12%, 0.2%, and 2%, applied two to four times a day, and delivered as a rinse, gel or in Vaseline. Thus, although topical application of CHX to the oral cavity of ventilated ICU patients in some cases appears to prevent VAP, the optimal concentration and frequency of application of this agent has not been validated. The present study was designed to determine if there was any difference in oral colonization with PRPs between once versus twice daily 0.12% CHX. There was no significant difference between once versus twice daily 0.12% CHX solution in terms of reducing oral colonization by PRPs, with the exception of *S. aureus*, whose numbers were reduced in dental plaque of individuals treated with CHX delivered once or twice a day.

Other environmental factors may also help explain the apparent limitation of CHX to reduce the number of PRPs in the oral cavity. The nurses at the hospital where the trial was conducted are well educated on the possible role of oral hygiene in VAP. The standardized oral care regime in place includes the debridement of the teeth and tongue by use of a suction toothbrush used twice a day together with swabbing with a peroxide rinse, swabbing with a peroxide rinse-impregnated swab every four hours, and use of a mouth moisturizer applied to the oral mucosa. Deep oropharyngeal suctioning is performed prior to major position changes and extubation, and as needed to remove oropharyngeal secretions that have pooled on top of the cuff of the endotracheal tube. This regime may have already reduced the number of organisms in the dental plaque to a level where additional reductions by CHX were not detectable. On the other hand, suctioning excess fluid at the time of application could have reduced the effect of CHX. Also, approximately 70% of the subjects enrolled in the present study were given antibiotics for reasons other than the development of VAP during the course of their ICU stay, for example for surgical prophylaxis or for treatment of another infection. Such exposure to antibiotics might have reduced the efficacy of CHX in this setting.

In summary, CHX applied topically once or twice a day to the oral cavity inhibited the numbers of *S. aureus *in the dental plaque of MV-ICU patients. An absence of an effect on the total number of target pathogens, or on Gram-negative species, is consistent with previous studies [13]. Perhaps an increase in the concentration of CHX or in the frequency of application would be more effective in reduction of pathogenic bacteria in oral biofilms. Additional large-scale clinical trials are required to determine the efficacy of CHX in the prevention of VAP in MV-ICU patients. Alternative approaches to reduce or eliminate PRPs from the oral cavity should also be studied, which may include other topical chemotherapeutic agents or frequent mechanical dislodgment of biofilms from the oral surfaces.

## Conclusions

The results of this trial show that oral topical CHX reduces the number of viable *S. aureus *in the dental plaque of MV-ICU patients. However, CHX did not reduce the total number or proportion of other target PRPs (*Pseudomonas*, *Acinetobacter *or enteric species) in dental plaque.

## Key messages

• Oral topical CHX reduces the number of viable *S. aureus *in the dental plaque of MV-ICU patients.

• CHX did not reduce the total number or proportion of other target PRPs in dental plaque, such as *Pseudomonas*, *Acinetobacter *or enteric species.

• No intra oral adverse events were noted, including mucositis or tooth staining.

• Although CHX reduced the number of cases of VAP compared with the placebo, the differences were found not to be statistically significant.

• No differences were noted for any outcome measured between groups treated once or twice a day with CHX.

## Abbreviations

ANOVA: analysis of variance; APACHE: Acute Physiology and Chronic Health Evaluation; BqBAL: blind quantitative bronchoalveolar lavage; cfu: colony forming units; CHX: chlorhexidine gluconate; CI: confidence interval; CPIS: clinical pulmonary infection score; ECMC: Erie County Medical Center; FiO_2_: fraction of inspired oxygen; HR: hazards ratio; MV-ICU: mechanically ventilated, intensive care unit; OR: odds ratio; PaO_2_: partial pressure of arterial oxygen; PRPs: potential respiratory bacterial pathogens; SID: subject Identification Numbers; VAP: ventilator-associated pneumonia.

## Competing interests

The authors declare that they have no competing interests.

## Authors' contributions

FAS conceived, designed and supervised the study, participated in analysis and interpretation of the data, drafted the manuscript. JY statistically analyzed that data and contributed to its interpretation. KR monitored subject inclusion and critically reviewed and revised the manuscript. JMM contributed to the study design, data interpretation and critically reviewed and revised the manuscript. AV acquired the data. SIO and KW organized and managed the data. All authors read and approved the final manuscript.
